# Absolute and relative grip strength as predictors of cancer: prospective cohort study of 445 552 participants in UK Biobank

**DOI:** 10.1002/jcsm.12863

**Published:** 2021-12-24

**Authors:** Solange Parra‐Soto, Jill P. Pell, Carlos Celis‐Morales, Frederick K. Ho

**Affiliations:** ^1^ Institute of Health and Wellbeing University of Glasgow Glasgow UK; ^2^ British Heart Foundation Cardiovascular Research Centre, Institute of Cardiovascular and Medical Sciences University of Glasgow Glasgow UK; ^3^ Human Performance Lab, Education, Physical Activity and Health Research Unit University Católica del Maule Talca Chile

**Keywords:** Cancer, Handgrip, Muscle mass

## Abstract

**Background:**

Reduced muscular strength, as measured by absolute grip strength, has been associated with increased risk of some site‐specific cancers. The ability of grip strength to predict other diseases may be affected by whether it is expressed in absolute or relative terms, but the evidence for cancer is scarce. This study compared the associations of absolute and relative grip strength with all‐cause and 15 site‐specific cancers.

**Methods:**

A prospective cohort study was undertaken using data from the UK Biobank. The exposure variable was grip strength, in absolute form (kilogrammes) and relative to weight, body mass index (BMI), height. and body fat mass. The outcome was incident cancer, at 15 sites and overall. Cox proportional hazard models were performed to study the associations.

**Results:**

This study included 445 552 participants, where 53.8% of the participants were women, with a mean (SD) age of 56.3 (8.11) years. During a median of 8.8 years follow‐up period, 48 886 (11.0%) patients were diagnosed with cancer. After adjusting for sociodemographic and lifestyle factors, as well as multiple testing, absolute grip strength was inversely and linearly associated with endometrial [hazard ratio (HR): 0.74, 95% confidence interval (CI): 0.69; 0.79, *P* value <0.001], gallbladder (HR: 0.81, 95% CI: 0.72; 0.92, *P* value = 0.001), liver (HR: 0.86, 95% CI: 0.79; 0.93, *P* value <0.001), kidney (HR: 0.93, 95% CI: 0.88; 0.99), and breast (HR: 0.93, 95% CI: 0.91; 0.96, *P* value = 0.031), as well as all‐cause cancer (HR: 0.97, 95% CI: 0.95; 0.98, *P* value <0.001). Eight cancer sites were inversely associated with HGS relative to weight and BMI: endometrium, liver, gallbladder, kidney, oesophagus, pancreas, colorectal, breast, and all‐cause cancer. Compared with absolute grip strength, grip strength relative to body fat mass had better discriminatory power for head and neck and breast cancer. Grip strength relative to BMI was marginally better than absolute grip strength in predicting stomach cancer.

**Conclusions:**

Grip strength was associated with risk of several site‐specific cancers and all‐cause cancer. Head and neck and breast cancers might be better predicted by relative grip strength.

## Introduction

There were 19.3 million new cancer cases in 2020,^1^ and by 2040, this number is expected to increase to 27.5 million.^2^ To alleviate the burden of cancer, several public health guidelines have been developed. The current physical activity guidelines include recommendations that aim to increase and maintain muscular strength across the life span.^3^

One of the most common muscle strength markers, in clinical and research settings, is handgrip strength (HGS) as it correlates well with overall strength.^4^, ^5^ HGS is a simple, non‐invasive and low‐cost method that has been associated with several chronic diseases and all‐cause mortality across different age groups.^6^, ^7^, ^8^ HGS has been associated with a range of health outcomes such as all‐cause mortality, cardiovascular diseases, and some site‐specific cancers (colorectal, lung, and breast) as well as all‐cause cancer.^5^, ^7^, ^9^, ^10^ However, evidence regarding the association of grip strength with cancer has been mainly restricted to absolute HGS, with limited and conflicting evidence available for site‐specific cancers.^4^, ^11^, ^12^

A meta‐analysis published in 2018, which included 309 413 participants and 9787 cases, found no association between HGS and overall cancer mortality. However, the categorization of strength and adjustment for covariates was heterogeneous between studies, and there was no differentiation between sites of cancer.^12^ The Prospective Urban Rural Epidemiology (PURE) study, which included data from 139 691 participants across 17 countries, reported that absolute HGS (per 5 kg reduction in HGS) was associated with increased overall cancer risk, especially in participants from high‐income countries.^13^ Some previous studies in UK Biobank reported associations of absolute HGS with all‐cause cancer, colorectal, lung, and breast cancer incidence and mortality.^11^ Whilst similar results were reported by Yates *et al*., the authors concluded that the association between absolute HGS and all‐cause cancer mortality was less consistent than other diseases.^14^ Individual study findings have also been inconsistent across cancer sites.^4^, ^12^, ^14^ Hence, Wu *et al*., in a meta‐analysis that included 42 studies, did not find an association between HGS and overall cancer [hazard ratio (HR): 0.89, 95% confidence interval (CI): 0.66–1.20].^4^

Studies have shown that relative HGS might be a better indicator for muscle weakness,^15^ as well as more predictive of cardiometabolic diseases.^16^ Because of these, there is yet a consensus on how HGS should be used in clinical practice.^17^ To our knowledge, all existing studies on HGS and cancer expressed HGS in absolute terms. The aims of this study, therefore, were to investigate the associations of HGS, expressed (i) in absolute terms (kilogrammes) and (ii) relative to anthropometric variables, with 15 cancer sites and all‐cause cancer and to compare risk prediction scores of HGS when differentially expressed.

## Methods

### Study design

Between April 2007 and December 2010, UK Biobank recruited approximately 502 000 participants, aged 37–73 years from the general population.^18^ Participants attended 1 of 22 assessment centres across England, Wales, and Scotland,^19^ where they completed a touch‐screen questionnaire, had physical measurements taken and provided biological samples, as described in detail elsewhere.^19^, ^20^ In this prospective population‐based study, 15 site‐specific cancers and all‐cause cancer incidence (fatal/non‐fatal) were the outcomes, HGS was the exposure variables; and socio‐demographic factors (age, ethnicity, area socioeconomic deprivation index), smoking status, sedentary behaviour, physical activity, height, diet (red and processes meat, oily fish, and alcohol), and multimorbidity were covariates. After excluding participants with cancer at baseline (*n* = 41 406), and with missing data for the exposure and covariates (*n* = 15 534), our sample was restricted to the 445 552 participants who had full data available.

### Procedure

Hospital admissions were identified via record linkage to Health Episode Statistics records for England (1 June 2020) and Wales (31 March 2017) and to Scottish Morbidity Records for Scotland (31 March 2017). The International Classification of Diseases, 10th revision (ICD‐10) was used to define the following 15 cancers: all cancers (C00‐C97, D37, and D48), and oral (C00‐C14), oesophageal (C15), stomach (C16), colorectal (C18, C19, and C20), liver (C22), gallbladder (C23), pancreatic (C25), lung (C34), kidney (C64‐C65), bladder (C67), breast (C50), endometrial (C54), cervical (C53), ovarian (C56), and prostate (C61) cancer. Of these, 10 cancer sites were used for men and women; one site was specific to men (prostate) and four to women (breast, endometrium, cervix, and ovary). Potential confounders were identified a priori based on established relationships with cancer and muscular strength. Area‐based socioeconomic status was derived from postcode of residence, using the Townsend score.^21^ Age at baseline was calculated from date of birth and date of baseline assessment. Medical history (physician diagnosis of depression, stroke, angina, heart attack, hypertension, cancer, diabetes, or long‐standing illness), ethnicity, smoking status (never, former, or current smoker), and female reproductive factors were collected from the self‐completed, baseline questionnaire. Dietary intake was collected via a food frequency questionnaire, with participants asked how many portions of red meat, processed meat, and fish they generally ate. Total time spent in discretionary sedentary behaviours was derived from the sum of self‐reported time spent driving, using a computer, and watching television. Anthropometric measurements, height, and weight were obtained during the baseline assessment by trained clinic staff using standard operating procedures and regularly calibrated equipment. Body fat was measured using the Tanita BC‐418 MA body composition analyser (fat mass divided by the total body mass). Further details of these measurements can be found in the UK Biobank online protocol (http://www.ukbiobank.ac.uk).

### Exposures

The HGS was assessed using a Jamar J00105 hydraulic hand dynamometer (Patterson Medical, Sutton‐in‐Ashfield, UK), and the mean of the right and left hand values, expressed as kilogramme, was used in the analysis, as reported elsewhere.^9^, ^22^ Five representations of HGS were analysed: (1) absolute HGS in kg, (2) HGS divided by height, (3) HGS divided by weight, (4) HGS divided by BMI, (5) HGS divided by body fat mass (BFM) in kilogramme. All these variables were standardized using sex‐specific mean and standard deviation of the whole sample ([X − Mean] ÷ SD).

### Statistical analyses

Continuous variables were summarized using mean and standard deviation, and categorical variables using frequencies and percentages. Non‐linear associations between HGS and cancer sites were visually explored using multivariable penalized cubic splines in Cox‐proportional hazard models.^23^ Penalized spline is a technique that balances data fit and smoothness.^24^ Spline curvature is penalized by the integrated second derivative. Knots were selected based on generalized cross‐validation and were equally spaced across the range of the exposure variable. The results were reported as hazard ratios (HRs) together with 95% confidence intervals (CIs). Analyses were adjusted for baseline age (at time of hand grip assessment), sex, ethnicity, Townsend deprivation index, height, smoking status, dietary intake (alcohol, red meat, oily fish, and processed meat), sedentary behaviour, physical activity, and comorbidities (longstanding illness, diabetes, hypertension, cardiovascular disease (CVD), cancer, and depression), as well as height when it was not included in the exposure. Additional covariates were added for breast, cervical, endometrial, and ovarian cancer: hormonal replacement (yes/no), contraceptive use (yes/no), and age at menarche. Finally, because of potentially inflated Type‐I errors due to multiple tests, we provided the adjusted *P* values (denoted as *P*_adj_) using Holm's method controlling family‐wise error rate.^25^

We calculated Harrell's C‐index (which estimates the probability of concordance between observed and predicted responses) to compare the discriminatory power of HGS markers.^26^ The proportional hazard assumption was checked by tests based on Schöenfeld residuals. All analyses were performed using R Statistical Software version 3.6.2 with the package survival. Statistical significance was set at α < 0.05.

### Patient involvement

No patients were involved in setting the research question or the outcome measures.

## Results

### Characteristics of the study population

There were 445 552 participants included in the analysis. The median follow‐up period was 8.8 years [IQR 7.9–9.6]. During the follow‐up period, 48 886 (11.0%) people developed cancer. *Table* 1 presents the characteristics of the study population. In summary, 53.8% of the cohort were women, the mean (SD) age was 56.3 (8.11) years, and the majority were white. People with lower HGS had a higher mean weight and waist circumference than those with moderate and higher strength, as well as a higher prevalence of obesity. No substantial differences were observed in lifestyle variables. However, more people in the lower strength group had been diagnosed with diabetes and hypertension and they had a higher multimorbidity count compared with people in the moderate and higher strength groups.

**Table 1:** Baseline characteristics by tertials of grip strength

	Lower HGS	Moderate HGS	Higher HGS	Overall
Sociodemographic				
*N* (%)	145 337 (32.6%)	152 701 (34.3%)	147 514 (33.1%)	445 552
Age, mean (SD)	58.6 (7.58)	56.7 (7.91)	53.4 (7.97)	56.3 (8.11)
Sex				
Females	79 127 (54.4%)	79 917 (52.3%)	80 794 (54.8%)	239 838 (53.8%)
Males	66 210 (45.6%)	72 784 (47.7%)	66 720 (45.2%)	205 714 (46.2%)
Townsend deprivation index				
Lower	43 016 (29.6%)	53 120 (34.8%)	54 111 (36.7%)	150 247 (33.7%)
Middle	47 056 (32.4%)	51 784 (33.9%)	50 158 (34.0%)	148 998 (33.4%)
Higher	55 265 (38.0%)	47 797 (31.3%)	43 245 (29.3%)	146 307 (32.8%)
Ethnicity				
White	135 052 (92.9%)	145 503 (95.3%)	140 908 (95.5%)	421 463 (94.6%)
Mixed	2420 (1.7%)	2081 (1.4%)	2175 (1.5%)	6676 (1.5%)
South Asian	4881 (3.4%)	2430 (1.6%)	1521 (1.0%)	8832 (2.0%)
Black	2593 (1.8%)	2240 (1.5%)	2333 (1.6%)	7166 (1.6%)
Chinese	391 (0.3%)	447 (0.3%)	577 (0.4%)	1415 (0.3%)
Anthropometric, mean (SD)				
Height (m)	1.7 (0.09)	1.7 (0.09)	1.7 (0.10)	1.7 (0.09)
Weight (kg)	81.4 (15.58)	77.8 (14.45)	75.2 (17.04)	78.1 (15.92)
Waist (cm)	94.8 (12.86)	90.1 (12.28)	85.9 (13.69)	90.2 (13.44)
Body fat percentage (%)	4.2 (9.55)	31.3 (8.15)	28.5 (6.75)	31.3 (8.54)
Body mass index (kg/m^2^)	29.2 (5.43)	27.2 (4.07)	25.8 (4.07)	27.4 (4.77)
BMI categories				
Underweight	443 (0.3%)	365 (0.2%)	1424 (1.0%)	2232 (0.5%)
Normal	30 431 (20.9%)	47 183 (30.9%)	67 814 (46.0%)	145 428 (32.6%)
Overweight	59 897 (41.2%)	72 249 (47.3%)	57 704 (39.1%)	189 850 (42.6%)
Obese	54 566 (37.5%)	32 904 (21.5%)	20 572 (13.9%)	108 042 (24.2%)
Lifestyle				
Smoking				
Never	78 642 (54.1%)	83 729 (54.8%)	83 776 (56.8%)	246 147 (55.2%)
Previous	51 866 (35.7%)	53 281 (34.9%)	47 681 (32.3%)	152 828 (34.3%)
Current	14 829 (10.2%)	15 691 (10.3%)	16 057 (10.9%)	46 577 (10.5%)
Alcohol intake				
Daily or almost daily	26 001 (17.9%)	32 400 (21.2%)	32 484 (22.0%)	90 885 (20.4%)
3–4 times a week	28 923 (19.9%)	36 618 (24.0%)	38 577 (26.2%)	104 118 (23.4%)
Once or twice a week	36 448 (25.1%)	39 956 (26.2%)	39 216 (26.6%)	115 620 (25.9%)
1–3 times a month	17 093 (11.8%)	16 848 (11.0%)	15 851 (10.7%)	49 792 (11.2%)
Special occasions only	21 104 (14.5%)	16 138 (10.6%)	13 119 (8.9%)	50 361 (11.3%)
Never	15 768 (10.8%)	10 741 (7.0%)	8267 (5.6%)	34 776 (7.8%)
Fruit and vegetable intake (portion/day), mean (SD)	2.0 (0.83)	2.0 (0.83)	2.0 (0.83)	2.0 (0.83)
Red meat (portion/week), mean (SD)	2.1 (1.49)	2.1 (1.43)	2.1 (1.42)	2.1 (1.45)
Processed meat (times/week), mean (SD)	1.9 (1.06)	1.9 (1.06)	1.8 (1.07)	1.9 (1.06)
Oily fish (times/week), mean (SD)	1.6 (0.95)	1.6 (0.92)	1.6 (0.91)	1.6 (0.93)
Sedentary time (h/day), mean (SD)	5.2 (2.36)	5.0 (2.24)	4.9 (2.23)	5.0 (2.28)
Physical activity (h/day), mean (SD)	2.1 (1.94)	1.8 (1.59)	1.7 (1.43)	1.8 (1.67)
Health				
Diabetes diagnosis				
No	133 364 (91.8%)	146 313 (95.8%)	144 063 (97.7%)	423 740 (95.1%)
Yes	11 973 (8.2%)	6388 (4.2%)	3451 (2.3%)	21 812 (4.9%)
Hypertension diagnosis				
No	95 572 (65.8%)	113 819 (74.5%)	119 971 (81.3%)	329 362 (73.9%)
Yes	49 765 (34.2%)	38 882 (25.5%)	27 543 (18.7%)	116 190 (26.1%)
Multimorbidty				
No illness	40 045 (27.6%)	57 711 (37.8%)	68 780 (46.6%)	166 536 (37.4%)
1 + illness	105 292 (72.4%)	94 990 (62.2%)	78 734 (53.4%)	279 016 (62.6%)

Data are shown in *n* (%) and mean (SD). SD, standard deviation. Data available 445 552.

#### Absolute HGS and incident cancers

**Figure 1:**
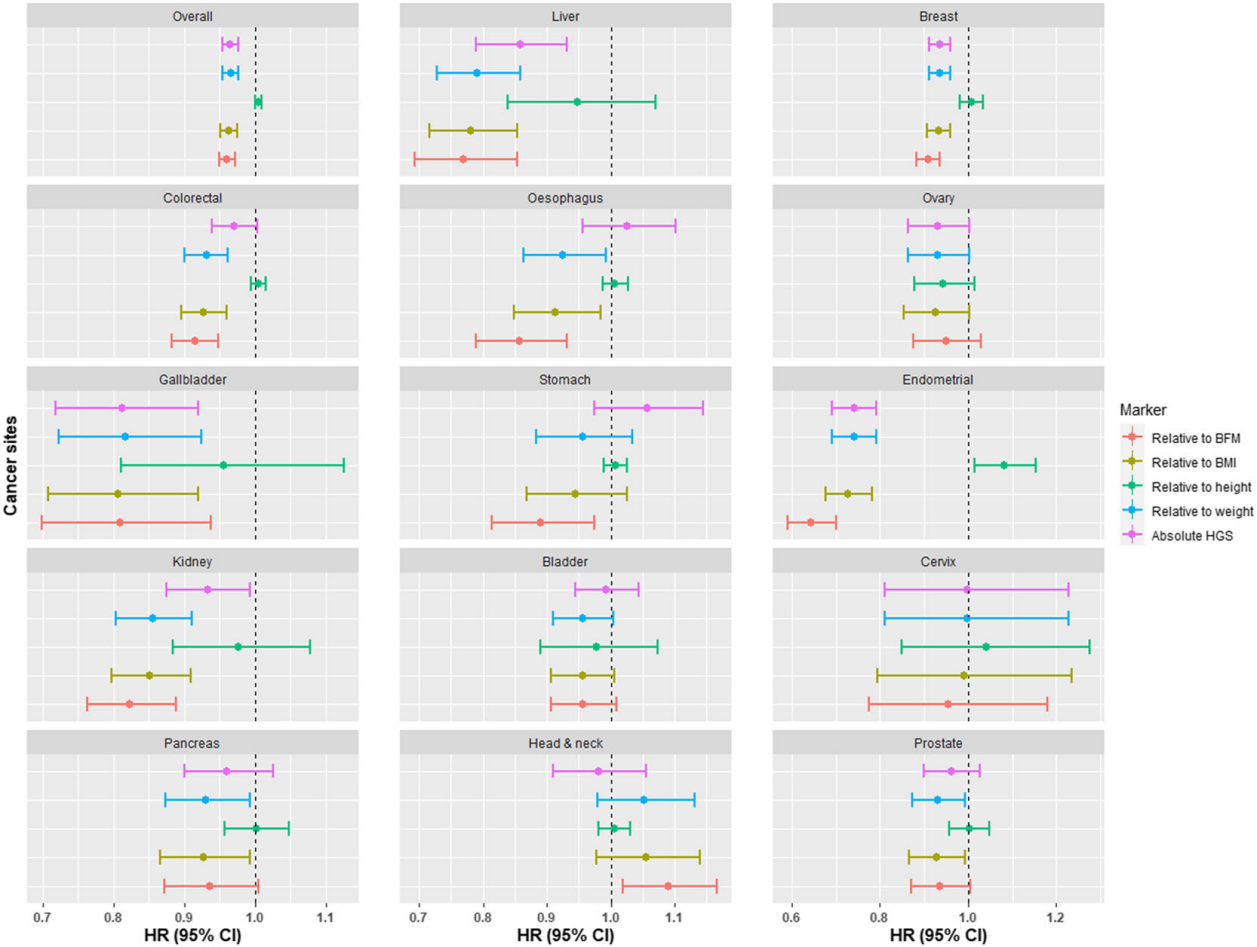
Association between relative grip strength and cancer incidence for 15 cancer. Data are presented in hazard ratio with 95% confidence intervals. Model was adjusted for age, sex, deprivation and ethnicity, height (except when height was part of the exposure), diet (red and process meat; oily fish and alcohol), smoking, physical activity sedentary behaviour, and comorbidity. Breast, cervix, endometrium and ovary also for age at menarche, hormonal replacement use, and contraceptive use. All *P* values were corrected for multiple testing by using the Holm's method. BMF, body fat mass; BMI, body mass index; HGS, hand grip strength.

Absolute HGS was inversely associated with five cancer sites: endometrium (HR: 0.74, 95% CI: 0.69; 0.79, *P* value <0.001), gallbladder (HR: 0.81, 95% CI: 0.72; 0.92, *P* value = 0.001), liver (HR: 0.86, 95% CI: 0.79; 0.93, *P* value <0.001), kidney (HR: 0.93, 95% CI: 0.88; 0.99, *P* value = 0.031), and breast (HR: 0.94, 95% CI: 0.91; 0.96, *P* value = <0.001), as well as all‐cause cancer (HR: 0.97, 95% CI: 0.95; 0.98, *P* value <0.001) (*Figure* 1 and *Table* S1). There was no strong evidence to suggest nonlinear associations (*Figure* S2).

#### Relative HGS and incident cancers

Eight cancer sites were inversely associated with HGS relative to weight and BMI: endometrium, liver, gallbladder, kidney, oesophagus, pancreas, colorectal, breast, and all‐cause cancer. The majority of these associations were linear (*Table* S1, *Figures* S2 and S3). The association patterns were similar for HGS relative to BFM, except that the association with stomach cancer was significant and with pancreatic cancer was not (*Table* S1 and *Figure* S5). HGS relative to height was inversely associated with only endometrial and lung cancer, as well as overall cancer (*Table* S1 and *Figure* S4). Prostate cancer was positively associated with almost all HGS markers (*Figure* 1 and *Table* S1) and head and neck cancer was positively associated with HGS relative to BFM.

### C‐index

*Table* 2 shows the Harrell's C‐indices for prediction of overall and site‐specific cancers. There were no significant differences in C‐indices between HGS expressed in absolute and relative terms for most cancer sites. However, HGS relative to BFM was better than absolute HGS in predicting head and neck and breast cancer. Also, HGS relative to BMI was better than absolute HGS at predicting stomach cancer.

**Table 2:** C‐indices of absolute and relative HGS in predicting cancer incidence

	Absolute HGS (95% CI)	Relative HGS (95% CI)	Difference (95% CI)	*P* value
Handgrip to weight				
Overall	0.6506 (0.6478; 0.6533)	0.6515 (0.6487; 0.6543)	−0.0009 (−0.0013; −0.0006)	<0.001
Head and neck	0.6774 (0.6580; 0.6959)	0.6753 (0.6558; 0.6943)	0.0020 (0.0001; 0.0039)	0.035
Oesophagus	0.7686 (0.7539; 0.7828)	0.7687 (0.7540; 0.7827)	−0.0001 (−0.0016; 0.0015)	0.945
Bladder	0.7742 (0.7642; 0.7840)	0.7741 (0.7641; 0.7839)	0.0001 (−0.0004; 0.0006)	0.742
Colorectal	0.6691 (0.6613; 0.6767)	0.6686 (0.6609; 0.6763)	0.0004 (−0.0005; 0.0013)	0.384
Gallbladder	0.6743 (0.6450; 0.7023)	0.6770 (0.6476; 0.7050)	−0.0026 (−0.0063; 0.0010)	0.154
Kidney	0.7111 (0.6973; 0.7243)	0.7091 (0.6953; 0.7226)	0.0019 (−0.0006; 0.0045)	0.135
Pancreas	0.6979 (0.6837; 0.7116)	0.6979 (0.6837; 0.7117)	0.0000 (−0.0016; 0.0016)	0.984
Stomach	0.7369 (0.7195; 0.7533)	0.7375 (0.7200; 0.7542)	−0.0006 (−0.0025; 0.0013)	0.552
Lung	0.8209 (0.8135; 0.8281)	0.8212 (0.8138; 0.8284)	−0.0003 (−0.0006; −0.0001)	0.003
Prostate	0.6809 (0.6757; 0.6861)	0.6807 (0.6755; 0.6859)	0.0002 (−0.0002; 0.0005)	0.332
Breast	0.5470 (0.5401; 0.5539)	0.5552 (0.5483; 0.5620)	−0.0082 (−0.0121; −0.0043)	<0.001
Endometrium	0.6497 (0.6339; 0.6653)	0.6497 (0.6338; 0.6652)	0.0001 (−0.0003; 0.0004)	0.761
Handgrip to height				
Overall	0.6502 (0.6475; 0.6530)	0.6515 (0.6487; 0.6543)	−0.0013 (−0.0016; −0.0009)	<0.001
Head and neck	0.6765 (0.6571; 0.6953)	0.6753 (0.6558; 0.6943)	0.0012 (0.0001; 0.0023)	0.039
Oesophagus	0.7686 (0.7539; 0.7826)	0.7687 (0.7540; 0.7827)	−0.0001 (−0.0005; 0.0003)	0.549
Bladder	0.7740 (0.7639; 0.7838)	0.7741 (0.7641; 0.7839)	−0.0001 (−0.0006; 0.0003)	0.517
Colorectal	0.6680 (0.6602; 0.6756)	0.6686 (0.6609; 0.6763)	−0.0007 (−0.0015; 0.0001)	0.104
Gallbladder	0.6678 (0.6384; 0.6961)	0.6770 (0.6476; 0.7050)	−0.0091 (−0.0170; −0.0013)	0.023
Kidney	0.7079 (0.6940; 0.7214)	0.7091 (0.6953; 0.7226)	−0.0013 (−0.0034; 0.0009)	0.250
Pancreas	0.5438 (0.5370; 0.5506)	0.5552 (0.5483; 0.5620)	−0.0114 (−0.0160; −0.0067)	<0.001
Stomach	0.6805 (0.6753; 0.6857)	0.6807 (0.6755; 0.6859)	−0.0002 (−0.0006; 0.0002)	0.284
Lung	0.7332 (0.7149; 0.7509)	0.7356 (0.7174; 0.7531)	−0.0024 (−0.0054; 0.0006)	0.111
Prostate	0.6970 (0.6829; 0.7109)	0.6979 (0.6837; 0.7117)	−0.0009 (−0.0024; 0.0007)	0.284
Breast	0.6319 (0.6163; 0.6470)	0.6497 (0.6338; 0.6652)	−0.0177 (−0.0293; −0.0062)	0.003
Endometrium	0.8212 (0.8138; 0.8283)	0.8212 (0.8138; 0.8284)	−0.0001 (−0.0003; 0.0002)	0.655
Handgrip to BMI				
Overall	0.6503 (0.6475; 0.6531)	0.6515 (0.6487; 0.6543)	−0.0012 (−0.0016; −0.0009)	<0.001
Head and neck	0.6774 (0.6580; 0.6959)	0.6753 (0.6558; 0.6943)	0.0020 (0.0002; 0.0039)	0.033
Oesophagus	0.7687 (0.7540; 0.7829)	0.7687 (0.7540; 0.7827)	0.0000 (−0.0016; 0.0015)	0.986
Bladder	0.7741 (0.7640; 0.7839)	0.7741 (0.7641; 0.7839)	0.0000 (−0.0006; 0.0005)	0.860
Colorectal	0.6686 (0.6608; 0.6762)	0.6686 (0.6609; 0.6763)	−0.0001 (−0.0010; 0.0009)	0.867
Gallbladder	0.6730 (0.6436; 0.7011)	0.6770 (0.6476; 0.7050)	−0.0040 (−0.0088; 0.0008)	0.102
Kidney	0.7099 (0.6961; 0.7232)	0.7091 (0.6953; 0.7226)	0.0008 (−0.0019; 0.0035)	0.572
Pancreas	0.5446 (0.5377; 0.5514)	0.5552 (0.5483; 0.5620)	−0.0106 (−0.0150; −0.0062)	<0.001
Stomach	0.6810 (0.6758; 0.6862)	0.6807 (0.6755; 0.6859)	0.0003 (0.0000; 0.0006)	0.046
Lung	0.7367 (0.7184; 0.7545)	0.7356 (0.7174; 0.7531)	0.0011 (−0.0021; 0.0043)	0.501
Prostate	0.6975 (0.6833; 0.7112)	0.6979 (0.6837; 0.7117)	−0.0004 (−0.0021; 0.0013)	0.619
Breast	0.6482 (0.6323; 0.6638)	0.6497 (0.6338; 0.6652)	−0.0015 (−0.0040; 0.0010)	0.232
Endometrium	0.8209 (0.8135; 0.8281)	0.8212 (0.8138; 0.8284)	−0.0003 (−0.0005; −0.0001)	0.003
Handgrip to BFM				
Overall	0.6506 (0.6478; 0.6533)	0.6515 (0.6487; 0.6543)	−0.0009 (−0.0013; −0.0006)	<0.001
Head and neck	0.6783 (0.6589; 0.6968)	0.6753 (0.6558; 0.6943)	0.0030 (0.0003; 0.0057)	0.031
Oesophagus	0.7692 (0.7545; 0.7837)	0.7687 (0.7540; 0.7827)	0.0006 (−0.0018; 0.0029)	0.638
Bladder	0.7742 (0.7641; 0.7839)	0.7741 (0.7641; 0.7839)	0.0000 (−0.0005; 0.0005)	0.947
Colorectal	0.6692 (0.6614; 0.6769)	0.6686 (0.6609; 0.6763)	0.0005 (−0.0006; 0.0017)	0.338
Gallbladder	0.6724 (0.6429; 0.7007)	0.6770 (0.6476; 0.7050)	−0.0046 (−0.0108; 0.0017)	0.152
Kidney	0.7113 (0.6976; 0.7244)	0.7091 (0.6953; 0.7226)	0.0022 (−0.0012; 0.0056)	0.201
Pancreas	0.5503 (0.5434; 0.5572)	0.5552 (0.5483; 0.5620)	−0.0049 (−0.0089; −0.0008)	0.019
Stomach	0.6809 (0.6757; 0.6861)	0.6807 (0.6755; 0.6859)	0.0002 (−0.0002; 0.0006)	0.408
Lung	0.7362 (0.7177; 0.7546)	0.7356 (0.7174; 0.7531)	0.0006 (−0.0032; 0.0044)	0.771
Prostate	0.6975 (0.6834; 0.7112)	0.6979 (0.6837; 0.7117)	−0.0004 (−0.0021; 0.0013)	0.667
Breast	0.6617 (0.6455; 0.6783)	0.6497 (0.6338; 0.6652)	0.0120 (0.0077; 0.0164)	<0.001
Endometrium	0.8208 (0.8134; 0.8281)	0.8212 (0.8138; 0.8284)	−0.0004 (−0.0007; −0.0001)	0.004

BMI, body mass index; CI, confidence interval; HGS, handgrip strength.

## Discussions

This paper reports the associations between HGS, in absolute and relative terms, and incident site‐specific and all‐cause cancer and explores the relative performance of these emerging risk markers in cancer risk prediction. Eight cancer sites were inversely associated with strength relative to weight, BMI, and BFM. Meanwhile, five cancer sites were inversely associated with absolute HGS. HGS expressed in relative terms modestly improved the prediction of head and neck, stomach, and breast cancer.

### Comparisons with other studies

The association patterns shown in this study are generally consistent with previous studies. HGS (per 5 kg decreases) was previously associated with lung, breast and colorectal cancer.^11^ In our study, both absolute and relative HGS, apart from HGS relative to height, were associated with breast cancer. Only relative HGS was associated with colorectal cancer and, whilst absolute HGS was associated with incident lung cancer in the partially adjusted models, it was not in the fully adjusted model including comorbidities.

To date, all studies have focused on absolute HGS, with equivocal results with most evidence relating to all‐cause cancer.^11^, ^13^ Gale *et al*. found a 19% decrease in overall cancer risk per 1‐SD increase of HGS,^27^ but García‐Hermoso *et al*. did not find the same association for cancer mortality (HR: 0.97, 95% CI: 0.92; 1.02).^12^ A previous large‐scale study showed a positive association between HGS and cancer mortality, but only in high‐income countries,^13^ consistent with our finding that, in the UK population, absolute and relative HGS were associated with lower risk of all‐cause cancer.

The HGS has been suggested as a good risk marker for other diseases, such as cardiovascular disease, irrespective of which HGS marker is used.^6^ HGS is a cheap and easy measure to incorporate into clinical practice.^28^ In our study, absolute HGS was a predictor of five site‐specific cancers as well as all‐cause cancer. Better prediction for some site‐specific cancers was achieved by using relative HGS. Further studies should explore the clinical utility of using absolute and relative HGS in the prevention and early detection of cancers.

The main finding of the current study was that when comparing numerous different ways to express HGS—absolute and relative to height, weight, BMI, and BFM—relative HGS only showed a modestly improvement in prediction of two groups of cancers. These findings could have important public health implications in terms of the operationalization of HGS in predicting cancer risk, which warrants further studies.^6^ This study demonstrates that the most basic form of reporting grip strength, namely in absolute units (kg), is largely sufficient for predicting cancer outcomes in clinical practice and further adjustment might not be needed.

### Limitations of this study

UK Biobank is not representative of the general population in terms of deprivation and lifestyle.^18^, ^19^ However, effect size estimates were generally consistent with population representative cohorts.^29^ As in all observational studies, residual confounding is possible, and association may not imply causation. Nonetheless, we minimized the risk of reverse causation using a two‐year landmark analysis. Even though UK Biobank has large sample size, there were small numbers of events for some site‐specific cancers which, therefore, might be underpowered.

## Conclusion

HGS was associated with a higher risk of several cancer sites and all‐cause cancer. HGS expressed in relative terms modestly improved the prediction of head and neck and breast cancers. Therefore, expressing grips strength in it most simple unit (kg) appears adequate for predicting most cancer outcomes.

## Conflict of interest

None to declare.

## Funding

UK Biobank was established and funded by the Wellcome Trust, Medical Research Council, Department of Health, Scottish Government, and the Northwest Regional Development Agency. It has also had funding from the Welsh Assembly Government and the British Heart Foundation. All authors had final responsibility for submission for publication. SPS was funded by the Chilean Government PhD scholarship programme.

## Supporting information


**Table S1.** Association between HGS z‐scores and cancer incidence
**Figure S1.** Association between absolute HGS and cancer incidence
**Figure S2.** Association between HGS relative to body weight and cancer incidence
**Figure S3.** Association between HGS relative to height and cancer incidence
**Figure S4.** Association between HGS relative to body mass index and cancer incidence
**Figure S5.** Association between HGS relative to body fat mass and cancer incidence
