# Supplementary material for: Absolute and relative grip strength as predictors of cancer: prospective cohort study of 445 552 participants in UK Biobank
Source: J Cachexia Sarcopenia Muscle. 2021 Dec 24;13(1):325–32. doi: 10.1002/jcsm.12863 (PMC8818619; doi:10.1002/jcsm.12863)
Supplement: Supplementary file 1 — Table S1. Association between HGS z‐scores and cancer incidence Figure S1. Association between absolute HGS and cancer incidence Figure S2. Association between HGS relative to body weight and cancer incidence Figure S3. Association between HGS relative to height and cancer incidence Figure S4. Association between HGS relative to body mass index and cancer incidence Figure S5. Association between HGS relative to body fat mass and cancer incidence [file JCSM-13-325-s001.docx]

**Supplementary: Parra-Soto S. et al. Absolute and relative grip strength as predictors of cancer: Prospective cohort study of 445,552 participants in UK Biobank**

Table S1: Association between HGS z-scores and cancer incidence

|  |  | Absolute HGS | | Relative to weight | | Relative to height | | Relative to BMI | | Relative to BFM | |
| --- | --- | --- | --- | --- | --- | --- | --- | --- | --- | --- | --- |
| Cancer | Total/events | HR (95% CI) | P | HR (95% CI) | P | HR (95% CI) | P | HR (95% CI) | P | HR (95% CI) | P |
| Overall | 437,170/37,085 | 0.97 (0.95; 0.98) | <0.001 | 0.97 (0.95; 0.98) | <0.001 | 1.00 (1.00; 1.01) | 0.013 | 0.96 (0.95; 0.97) | <0.001 | 0.96 (0.95; 0.97) | <0.001 |
| Head & neck | 442,799/848 | 0.98 (0.91; 1.06) | 0.597 | 1.05 (0.98; 1.13) | 0.172 | 1.01 (0.98; 1.03) | 0.679 | 1.06 (0.98; 1.14) | 0.164 | 1.09 (1.02; 1.17) | 0.013 |
| Oesophagus | 442,778/954 | 1.03 (0.96; 1.10) | 0.478 | 0.93 (0.86; 0.99) | 0.029 | 1.01 (0.99; 1.03) | 0.532 | 0.91 (0.85; 0.98) | 0.017 | 0.86 (0.79; 0.93) | <0.001 |
| Liver | 442,849/695 | 0.86 (0.79; 0.93) | <0.001 | 0.79 (0.73; 0.86) | <0.001 | 0.95 (0.84; 1.07) | 0.380 | 0.78 (0.72; 0.85) | <0.001 | 0.77 (0.69; 0.85) | <0.001 |
| Stomach | 442,819/757 | 1.06 (0.97; 1.14) | 0.181 | 0.96 (0.88; 1.03) | 0.253 | 1.01 (0.99; 1.02) | 0.461 | 0.94 (0.87; 1.03) | 0.174 | 0.89 (0.81; 0.97) | 0.011 |
| Pancreas | 442,796/1,154 | 0.96 (0.90; 1.03) | 0.229 | 0.93 (0.87; 0.99) | 0.030 | 1.00 (0.96; 1.05) | 0.950 | 0.93 (0.87; 0.99) | 0.030 | 0.94 (0.87; 1.01) | 0.070 |
| Lung | 442,497/3,345 | 0.97 (0.93; 1.01) | 0.092 | 1.01 (0.97; 1.05) | 0.671 | 0.93 (0.89; 0.98) | 0.007 | 1.00 (0.97; 1.04) | 0.837 | 1.02 (0.98; 1.06) | 0.240 |
| Gallbladder | 442,885/316 | 0.81 (0.72; 0.92) | 0.001 | 0.82 (0.72; 0.92) | 0.001 | 0.96 (0.81; 1.13) | 0.588 | 0.81 (0.71; 0.92) | 0.001 | 0.81 (0.70; 0.94) | 0.005 |
| Bladder | 442,608/1,984 | 0.99 (0.94; 1.04) | 0.768 | 0.96 (0.91; 1.00) | 0.068 | 0.98 (0.89; 1.07) | 0.631 | 0.96 (0.91; 1.01) | 0.084 | 0.96 (0.91; 1.01) | 0.105 |
| Kidney | 442,765/1,201 | 0.93 (0.88; 0.99) | 0.031 | 0.86 (0.80; 0.91) | <0.001 | 0.98 (0.88; 1.08) | 0.633 | 0.85 (0.80; 0.91) | <0.001 | 0.82 (0.76; 0.89) | <0.001 |
| Colorectal | 442,160/4,457 | 0.97 (0.94; 1.00) | 0.081 | 0.93 (0.90; 0.96) | <0.001 | 1.00 (0.99; 1.02) | 0.403 | 0.93 (0.90; 0.96) | <0.001 | 0.91 (0.88; 0.95) | <0.001 |
| Prostate | 203,050/7,327 | 1.03 (1.01; 1.06) | 0.012 | 1.04 (1.02; 1.07) | 0.001 | 1.00 (0.97; 1.03) | 0.883 | 1.05 (1.02; 1.07) | 0.001 | 1.04 (1.01; 1.06) | 0.007 |
| Breast | 237,735/6,776 | 0.94 (0.91; 0.96) | <0.001 | 0.94 (0.91; 0.96) | <0.001 | 1.01 (0.98; 1.03) | 0.629 | 0.93 (0.91; 0.96) | <0.001 | 0.91 (0.88; 0.93) | <0.001 |
| Ovary | 238,853/870 | 0.93 (0.86; 1.00) | 0.062 | 0.93 (0.86; 1.00) | 0.062 | 0.94 (0.88; 1.01) | 0.112 | 0.93 (0.85; 1.00) | 0.058 | 0.95 (0.88; 1.03) | 0.199 |
| Endometrium | 238,841/1,092 | 0.74 (0.69; 0.79) | <0.001 | 0.74 (0.69; 0.79) | <0.001 | 1.08 (1.01; 1.15) | 0.016 | 0.73 (0.68; 0.78) | <0.001 | 0.64 (0.59; 0.70) | <0.001 |
| Cervix | 238,988/108 | 1.00 (0.81; 1.23) | 0.982 | 1.00 (0.81; 1.23) | 0.982 | 1.04 (0.85; 1.28) | 0.704 | 0.99 (0.79; 1.23) | 0.934 | 0.96 (0.77; 1.18) | 0.672 |

_Data are presented in hazard ratio with 95% confidence intervals. Model was adjusted for age, sex, deprivation and ethnicity, height (except in HGS height), diet (red & process meat, fruits & vegetables, oily fish & alcohol), smoking and sedentary behaviour and comorbidity. Breast, cervix, endometrium and ovary also for age menarche, hormonal replacement use and contraceptive use. All P-values were corrected for multiple testing by using the Holm’s method. HGS: hand grip strength, BMI: body mass index, BMF: body fat mass, FFM: free fat mass. In red and bold significant results after multiple testing._


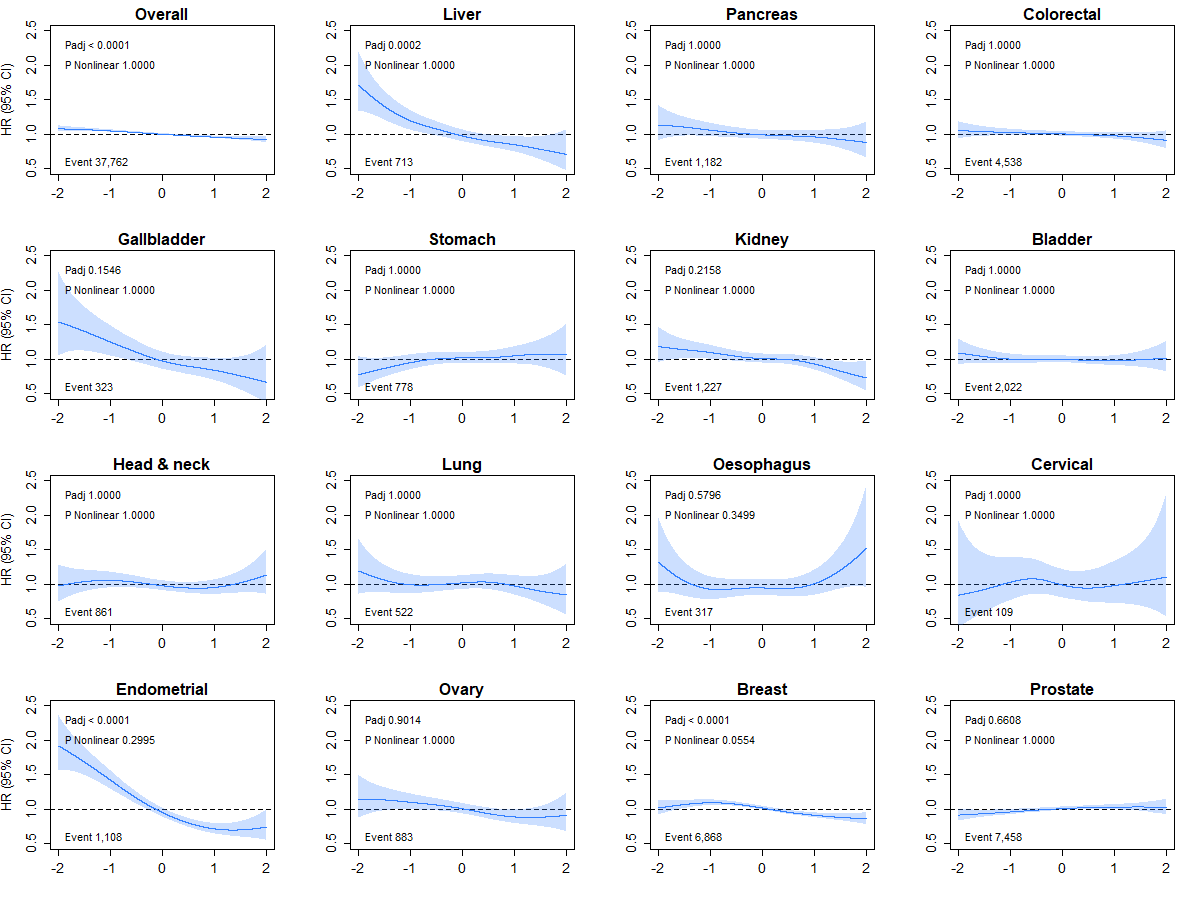


**Figure S1:** Association between absolute HGS and cancer incidence

Data are presented in hazard ratio with 95% confidence intervals. Analyses were adjusted for age, sex, deprivation, ethnicity, height (except in HGS relative to height), diet (red & process meat, fruits & vegetables, oily fish & alcohol), smoking, sedentary behaviour and comorbidity. For breast, cervix, endometrium, and ovary cancer also hormonal replacement (yes/no), contraceptive use (yes/no) and age menarche. All P-values were corrected for multiple testing by using the Holm’s method. HGS: hand grip strength, BMI: body mass index, BMF: body fat mass, FFM: free fat mass.


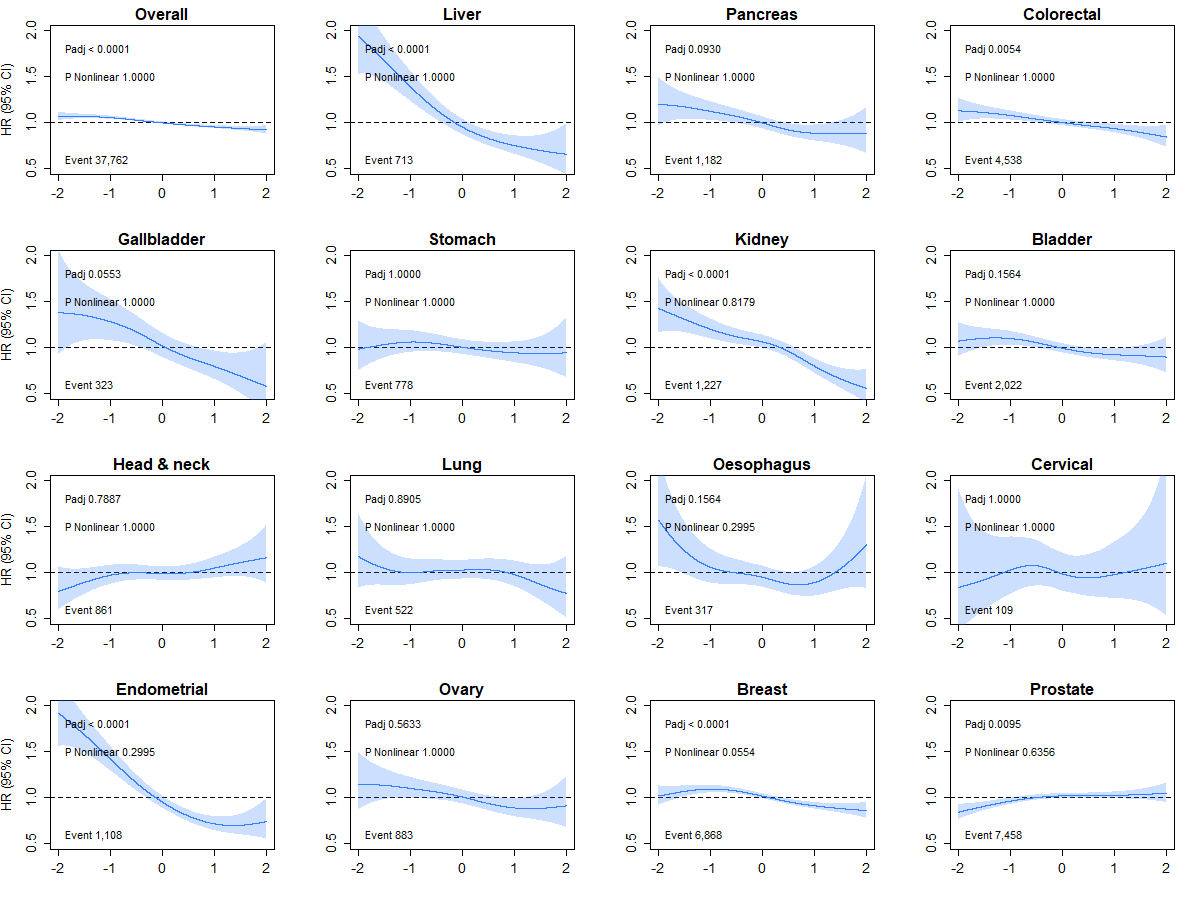


**Figure S2:** Association between HGS relative to body weight and cancer incidence

Data are presented in hazard ratio with 95% confidence intervals. Analyses were adjusted for age, sex, deprivation, ethnicity, height (except in HGS height), diet (red & process meat, fruits & vegetables, oily fish & alcohol), smoking, sedentary behaviour and comorbidity. For breast, cervix, endometrium, and ovary cancer also hormonal replacement (yes/no), contraceptive use (yes/no) and age menarche. All P-values were corrected for multiple testing by using the Holm’s method. HGS: hand grip strength, BMI: body mass index, BMF: body fat mass, FFM: free fat mass.


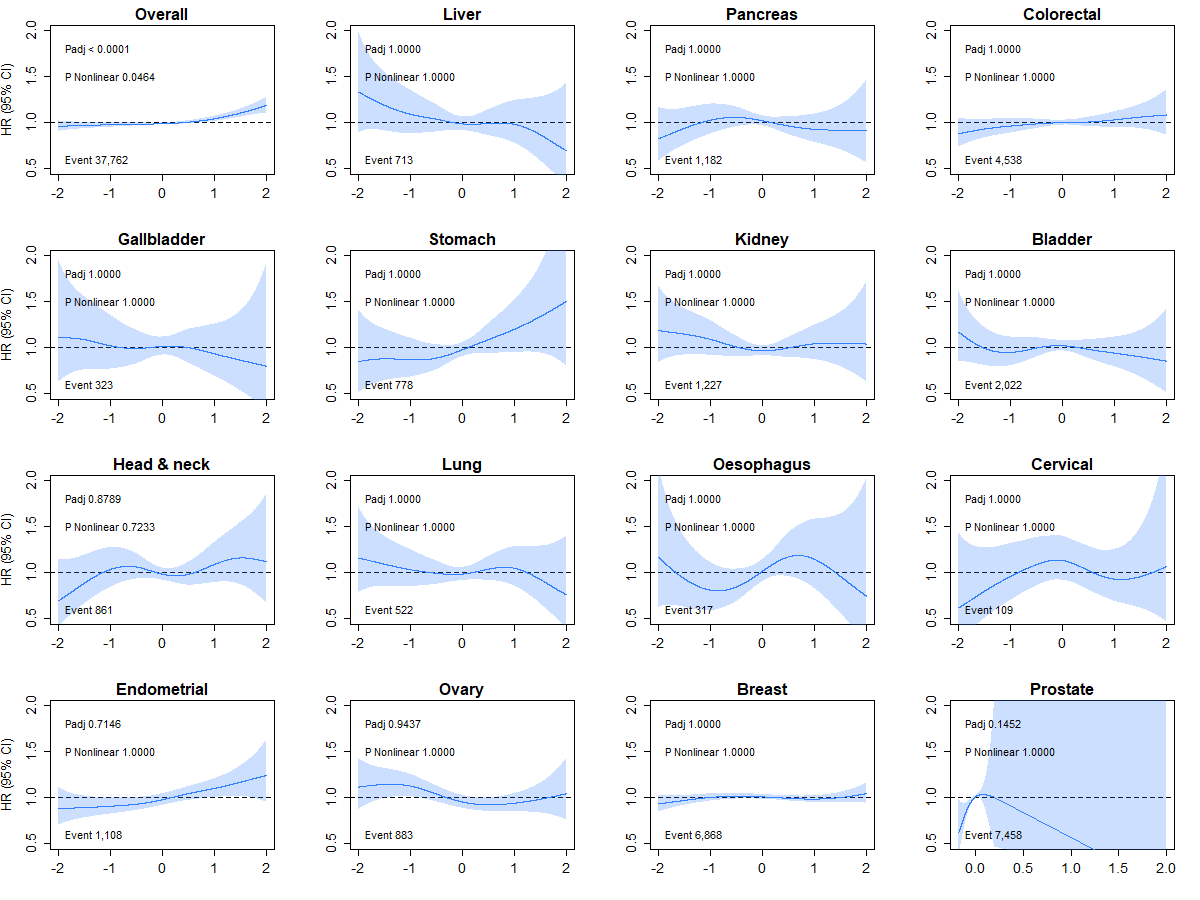


**Figure S3:** Association between HGS relative to height and cancer incidence

Data are presented in hazard ratio with 95% confidence intervals. Analyses were adjusted for age, sex, deprivation, ethnicity, height (except in HGS height), diet (red & process meat, fruits & vegetables, oily fish & alcohol), smoking, sedentary behaviour and comorbidity. For breast, cervix, endometrium, and ovary cancer also hormonal replacement (yes/no), contraceptive use (yes/no) and age menarche. All P-values were corrected for multiple testing by using the Holm’s method. HGS: hand grip strength, BMI: body mass index, BMF: body fat mass, FFM: free fat mass.


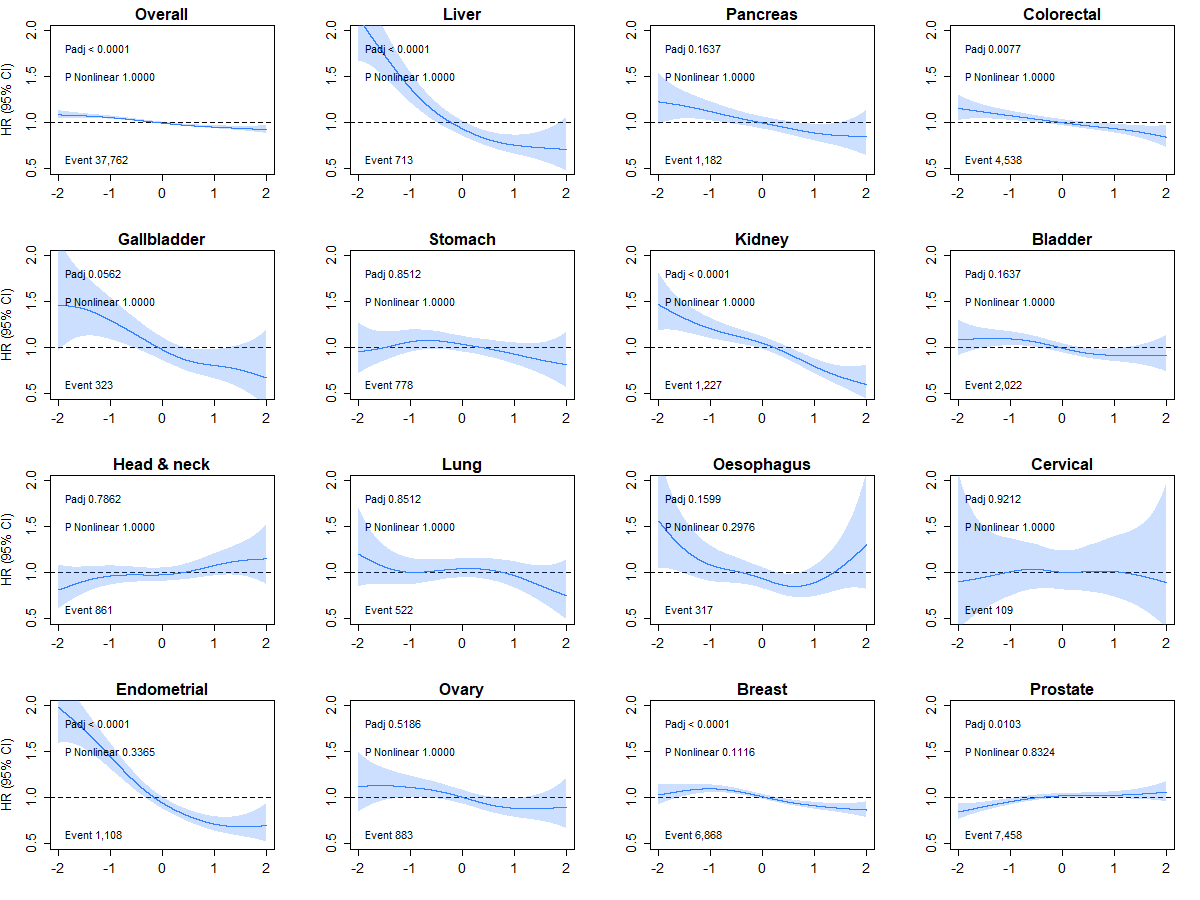


**Figure S4:** Association between HGS relative to body mass index and cancer incidence

Data are presented in hazard ratio with 95% confidence intervals. Analyses were adjusted for age, sex, deprivation, ethnicity, height (except in HGS height), diet (red & process meat, fruits & vegetables, oily fish & alcohol), smoking, sedentary behaviour and comorbidity. For breast, cervix, endometrium, and ovary cancer also hormonal replacement (yes/no), contraceptive use (yes/no) and age menarche. All P-values were corrected for multiple testing by using the Holm’s method. HGS: hand grip strength, BMI: body mass index, BMF: body fat mass, FFM: free fat mass.


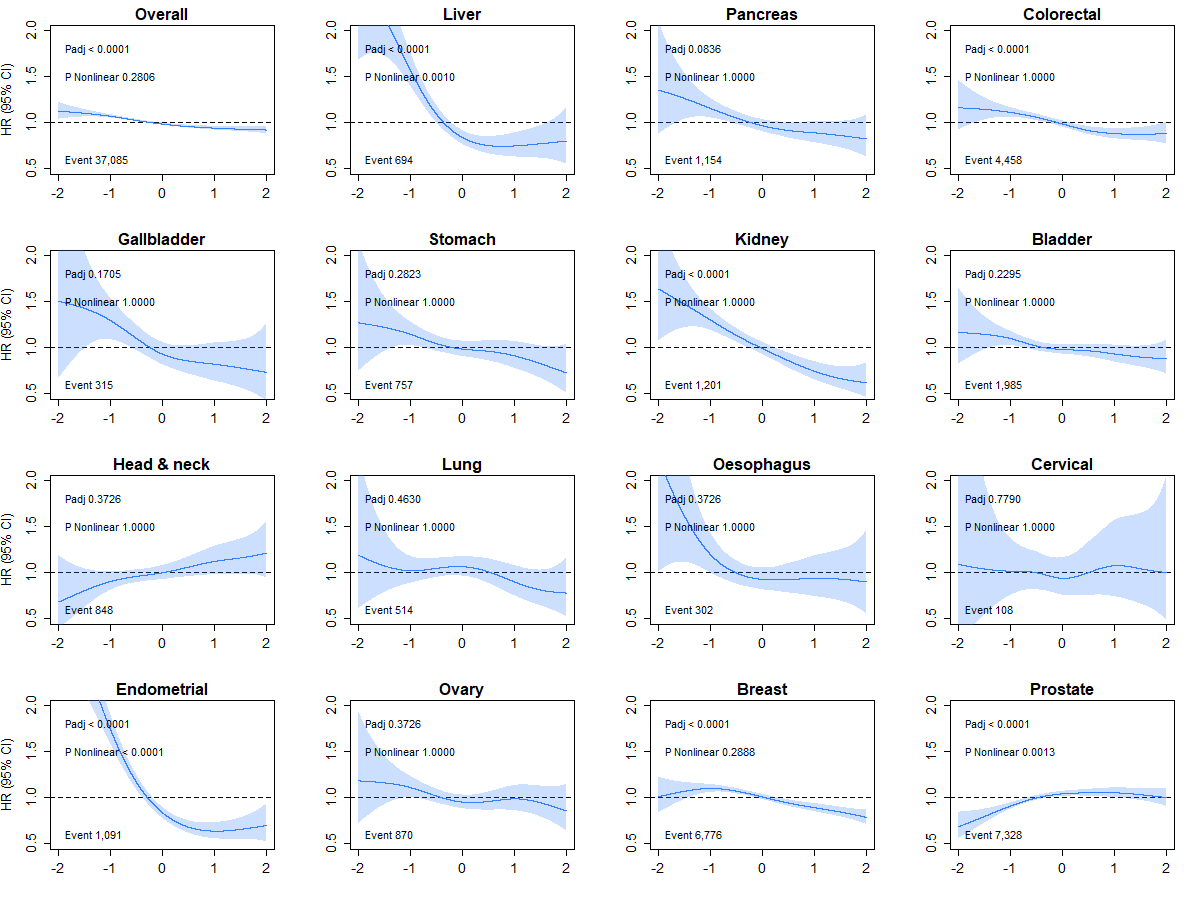


**Figure S5:** Association between HGS relative to body fat mass and cancer incidence

Data are presented in hazard ratio with 95% confidence intervals. Analyses were adjusted for age, sex, deprivation, ethnicity, height (except in HGS height), diet (red & process meat, fruits & vegetables, oily fish & alcohol), smoking, sedentary behaviour and comorbidity. For breast, cervix, endometrium, and ovary cancer also hormonal replacement (yes/no), contraceptive use (yes/no) and age menarche. All P-values were corrected for multiple testing by using the Holm’s method. HGS: hand grip strength, BMI: body mass index, BMF: body fat mass, FFM: free fat mass.
